# Supplementary material for: Agonistic CD40 therapy induces tertiary lymphoid structures but impairs responses to checkpoint blockade in glioma
Source: Nat Commun. 2021 Jul 5;12:4127. doi: 10.1038/s41467-021-24347-7 (PMC8257767; doi:10.1038/s41467-021-24347-7)
Supplement: Supplementary file 1 — Supplementary information [file 41467_2021_24347_MOESM1_ESM.pdf]

Agonistic CD40 therapy induces tertiary lymphoid structures but impairs responses to checkpoint blockade in glioma

Supplementary information

Supplementary Table 1. Histological characteristics and WHO classification of the human glioma samples included in the cohort. All samples were de-novo gliomas collected from treatment-naïve patients. The table also reports TLS presence and classification for each patient. Abbreviations: mut (mutation); co-del (co-deletion); NA (not assessed).

Patient ID	IDH1 mut	1p/19q co-del	ATRX mut	p53 mut	Histological diagnosis (WHO)	Grade	Type of section	TLS presence	Immature TLS	Organized TLS
**1**	-	-	-	-	Astrocytoma	II	En block, large	-	0	0
**2**	+	-	+	-	Astrocytoma	II	En block, large	-	0	0
**3**	+	-	+	-	Astrocytoma	II	En block, large	-	0	0
**4**	+	+	-	-	Oligodendroglioma	II	En block, large	-	0	0
**5**	+	+	-	-	Oligodendroglioma	II	En block, large	+	0	3
**6**	+	+	-	-	Oligodendroglioma	II	En block, large	+	3	0
**7**	-	-	-	-	Anaplastic astrocytoma	III	En block, large	-	0	0
**8**	+	+	-	-	Anaplastic oligodendroglioma	III	En block, large	+	6	1
**9**	+	+	-	-	Anaplastic oligodendroglioma	III	En block, large	-	0	0
**10**	+	+	-	-	Anaplastic oligodendroglioma	III	En block, large	-	0	0
**11[#]**	-	-	-	-	Glioblastoma	IV	En block, large	+	6	0
**12**	-	-	NA	-	Glioblastoma	IV	Large biopsy	-	0	0
**13**	-	-	NA	+	Glioblastoma	IV	Large biopsy	+	1	4
**14**	-	-	NA	-	Glioblastoma	IV	Large biopsy	-	0	0
**15**	-	-	NA	+	Glioblastoma	IV	Large biopsy	-	0	0
**16**	-	-	NA	+	Glioblastoma	IV	Large biopsy	+	1	3
**17**	-	-	NA	+	Glioblastoma	IV	Large biopsy	-	0	0
**18**	-	-	NA	+	Glioblastoma	IV	Large biopsy	-	0	0
**19**	-	-	NA	+	Glioblastoma	IV	Large biopsy	-	0	0
**20**	-	-	NA	+	Glioblastoma	IV	Large biopsy	-	0	0
**21**	-	-	NA	-	Glioblastoma	IV	Large biopsy	+	1	0
**22**	-	-	NA	-	Glioblastoma	IV	Large biopsy	+	1	0
**23**	-	-	NA	-	Glioblastoma	IV	Large biopsy	+	0	1
**24**	-	-	NA	-	Glioblastoma	IV	Large biopsy	+	2	0
**25***	-	-	-	-	Glioblastoma	IV	Large biopsy	+	0	4
**26**	-	-	-	-	Glioblastoma	IV	Large biopsy	-	0	0

[#]Patient 11: this patient was also afflicted with chronic lymphocytic leukemia

*Patient 25: samples collected from a primary and a secondary surgery were available. TLS were found in both cases.

Supplementary Table 2. Mouse primer sequences used for real-time quantitative PCR.

Target	Primer sequence (5' - 3')	
Cxcl13	Fw	CATAGATCGGATTCAAGTTACGCC
	Rs	TCTTGGTCCAGATCACAACTTCA
Ccl19	Fw	ACCACACTAAGGGGCTATCAG
	Rs	TTCTTCAGTCTTCGGATGATGC
Ccl21	Fw	GCTGCAAGAGAACTGAACAGACA
	Rs	CGTGAACCACCCAGCTTGA
Ccl22	Fw	AGGTCCCTATGGTGCCAATGT
	Rs	CGGCAGGATTTTGAGGTCCA
Il10	Fw	GCTCTTACTGACTGGCATGAG
	Rs	CGCAGCTCTAGGAGCATGTG
Lgals1	Fw	CAAGCTGCCAGACGGACAT
	Rs	AGGCCACGCACTTAATCTTGA
Lta	Fw	GCATCTTCTAAGCCCTGGGGG
	Rs	TGTCATGTGGAGGACCTGCTGTG
Ltb	Fw	GTTCAACAGCTGCCAAAGGG
	Rs	CATCCAAGCGCCTATGAGGT
Tgfβ	Fw	CTCCCGTGGCTTCTAGTGC
	Rs	GCCTTAGTTTGGACAGGATCTG
Tnfsf14	Fw	AGCAGCACATCTTACAGGAGC
	Rs	AGCTGCACTTTGGAGTACACA

Supplementary Table 3. List of antibodies used for immunostaining of mouse vibratome sections, cryosections and tissue for laser microdissection (LMD).

	Marker	Clone	Fluorochrome	Dilution	Company	Identifier (Cat # and RRID)
**Used for LMD**	B220	RA3-6B2	PE	1 : 100	Biolegend	# 103207 RRID:AB_312992
	CD45	30-F11	AF488	1 : 100	Biolegend	# 103122 RRID:AB_493531
**Used for cryo- or vibratome sections**	Anti-rat	polyclonal	AF488	1 : 500	Life Technologies	# A-21208 RRID:AB_2535794
	CD11b	M1/70	APC	1 : 100	Biolegend	# 101211 RRID:AB_312794
	CD11c	N418	-	1 : 100	Abcam	# ab33483 RRID:AB_726084
	CD19	polyclonal	-	1 : 100	Abcam	# ab227019 RRID:n/a
	CD21	SC0681	-	1 : 100	Thermo Fisher	# MA5-32227 RRID:AB_2809513
	CD23	PA5-79242	-	1 : 100	Thermo Fisher	# PA5-79242 RRID:AB_2746358
	CD3	17A2	BV421	1 : 100	BD	# 564008 RRID:AB_2732058
	CD31	2H8	-	1 : 100	Thermo Fisher	# MA3105 RRID:AB_223592
	CD35	8C12	DyLight 350	1 : 50	Novus Biologicals	# NBP2-52668UV RRID:n/a
	CD45	30-F11	APC	1 : 100	Biolegend	# 103112 RRID:AB_312977
	CD62L	MEL-14	AF647	1 : 100	Biolegend	# 104421 RRID:AB_493379
	ColIV	polyclonal	-	1 : 100	Abcam	# ab6586 RRID:AB_305584
	FN	polyclonal	-	1 : 100	Abcam	# ab2413 RRID:AB_2262874
	FoxP3	150D	AF647	1 : 100	Biolegend	#320013 RRID:AB_439749
	F4/80	BM8	PeCy5	1 : 100	Biolegend	# 123111 RRID:AB_893494
	IgG- F'2 fragment specific	polyclonal	AF647	1 : 100	JIR	# 115-606-006 RRID:AB_2338922
	Ki67	SP6	-	1 : 100	Abcam	# ab16667 RRID:AB_302459

Supplementary Table 4. List of antibodies and dyes used to characterize T cells and antigen presenting cells (APCs) in the tumor microenvironment (TME) by flow cytometry.

	Marker	Clone	Fluorochrome	Dilution	Company	Identifier (Cat # and RRID)
**17-color panel used to characterize T cells**	CD45	30-F11	BV510	1 : 100	BD	# 563891, RRID:AB_2734134
	CD3e	145-2C11	BV421	1 : 100	BD	# 562600, RRID:AB_11153670
	CD8	53-6.7	APC-Cy7	1 : 100	BD	# 557654, RRID:AB_396769
	CD4	GK1.5	BUV496	1 : 100	BD	# 612952, RRID:AB_2813886
	CD44	IM7	FITC	1 : 100	Biolegend	# 103022, RRID:AB_493685
	CD62L	MEL-14	PE-Cy7	1 : 100	BD	# 565535, RRID:AB_2739286
	CD69	H1.2F3	BB700	1 : 100	BD	# 566500, RRID:AB_2744450
	CD127	A7R34	PE-Cy5	1 : 100	Biolegend	# 135016, RRID:AB_1937261
	PD-1	29F.1A12	BV785	1 : 100	Biolegend	# 135225, RRID:AB_2563680
	TIM-3	RMT3-23	BV605	1 : 100	Biolegend	# 119721, RRID:AB_2616907
	LAG-3	C9B7W	BV711	1 : 100	BD	# 563179, RRID:AB_2737398
	Ki67	16A8	PE dazzle594	1 : 100	Biolegend	# 652428, RRID:AB_2632696
	CXCR5	L138D7	BV650	1 : 100	Biolegend	# 145517, RRID:AB_2562453
	KLRG1	2F1	BUV737	1 : 100	BD	# 741812, RRID:AB_2871150
	Foxp3	150D	AF647	1 : 100	Biolegend	#320013, RRID:AB_439749
	CD25	3C7	PE	1 : 100	Biolegend	# 101904, RRID:AB_312847
	LiveDead	-	Fixable viability dye 700	1 : 2500	BD	# 564997
**17-color panel used to characterize APCs**	CD45	30-F11	BV510	1 : 100	BD	# 563891, RRID:AB_2734134
	IL10	JES5-16E3	PE dazzle594	1 : 50	Biolegend	# 505034, RRID:AB_2566329
	CD19	6D5	APC-Cy7	1 : 50	Biolegend	# 115512, RRID:AB_313647
	IA/IE	M5/114.15.2	BB700	1 : 200	BD	# 746197, RRID:AB_2743544
	CD11b	M1/70	BUV395	1 : 100	BD	# 565976, RRID:AB_2721166
	Ly6G	1A8	BV421	1 : 100	BD	# 562737, RRID:AB_2737756
	CD11c	N418	PE-Cy5	1 : 100	Biolegend	# 117316, RRID:AB_493566
	CX3CR1	SA011F11	BV650	1 : 200	Biolegend	# 149033, RRID:AB_2565999
	Ly-6C	HK1.4	BV785	1 : 200	Biolegend	# 128041, RRID:AB_2565852
	CD103	M290	BV711	1 : 50	BD	743791, RRID:AB_2741759
	F4/80	6F12	BV605	1 : 100	BD	# 744337, RRID:AB_2742164
	PD-L1	10F.9G2	PE	1 : 100	Biolegend	124308, RRID:AB_2073556
	IL-12 (p40/p70)	C15.6	APC	1 : 50	BD	# 554480, RRID:AB_398560
	iNOS	CXNFT	A488	1 : 100	eBioscience	# 53-5920-82, RRID:AB_2574423
	Arginase I	A1exF5	PE-Cy7	1 : 100	eBioscience	# 25-3697-80, RRID:AB_2734840
	CD86	GL-1	BUV737	1 : 100	BD	# 741737, RRID:AB_2871107
	LiveDead	-	Fixable viability dye 700	1 : 2500	BD	# 564997

Supplementary Table 5. List of additional antibodies and dyes used for flow cytometry or FACS.

	Marker	Clone	Fluorochrome	Dilution	Company	Identifier (Cat # and RRID)
**Other antibodies used for flow cytometry or FACS**	CD45	30-F11	FITC	1 : 100	Biolegend	# 103107, RRID:AB_312972
	B220	RA3-6B2	AF488	1 : 100	Biolegend	# 103225, RRID:AB_389308
	B220	RA3-6B2	PE	1 : 100	Biolegend	# 103208, RRID:AB_312993
	B220	RA3-6B2	APCCy7	1 : 100	Biolegend	# 103223, RRID:AB_313006
	CD1d	1B1	BV421	1 : 100	BD	# 562712, RRID:AB_2737739
	CD19	6D5	APC	1 : 100	Biolegend	# 115512, RRID:AB_313647
	CD19	1D3	PerCP-Cy5.5	1 : 100	BD	# 561113, RRID:AB_10563071
	CD4	GK1.5	PerCP	1 : 100	Biolegend	# 100432, RRID:AB_893323
	CD4	RM4-5	AF488	1 : 100	Biolegend	# 100529, RRID:AB_389303
	CD5	53-7.3	PE-Cy5	1 : 100	Biolegend	# 100610, RRID:AB_312739
	CD107a	1D4B	PECy7	1 : 100	Biolegend	# 121620, RRID:AB_2562147
	CD69	H1.2F3	PE	1 : 100	BD	# 561932, RRID:AB_10897992
	CD69	H1.2F3	APC	1 : 100	Biolegend	# 104514, RRID:AB_492843
	CD11b	M1/70	APC	1 : 100	Biolegend	# 101212, RRID:AB_312795
	CD19	6D5	FITC	1 : 100	Biolegend	# 115506, RRID:AB_313641
	CD3	145-2C11	PerCP-Cy5.5	1 : 100	Thermo Fisher	# 45-0031-80, RRID:AB_906226
	CD8	53-6.7	APC-Cy7	1 : 100	BD	# 557654, RRID:AB_396769
	IFNγ	XMG1.2	PE	1 : 50	BD	# 562303, RRID:AB_11153140
	LiveDead	-	Zombie Aqua	1 : 1000	Biolegend	# 423102

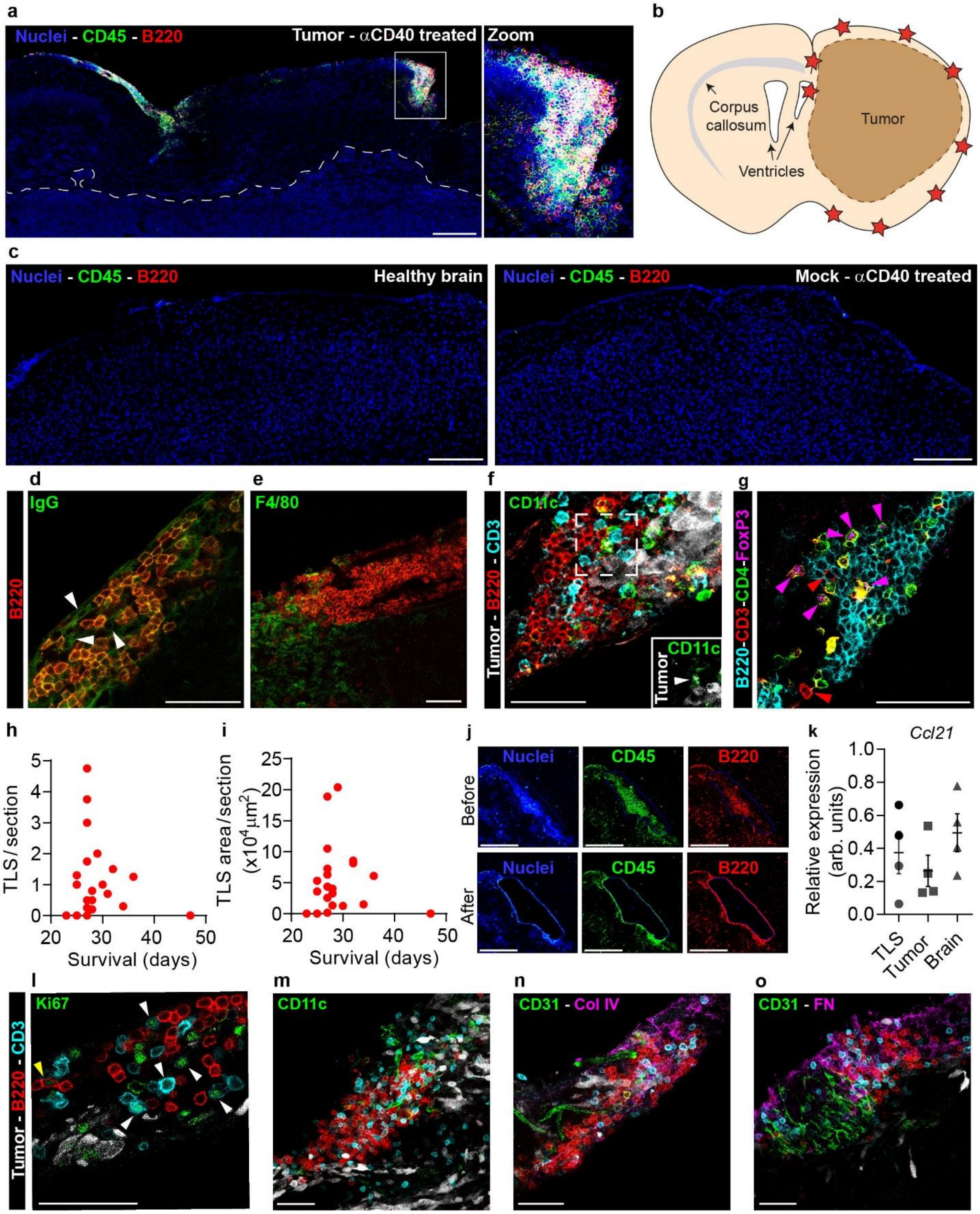

Supplementary Fig. 1: Induction of tertiary lymphoid structures (TLS) in murine glioma models.

(a) Immunofluorescent staining of dense CD45$^+$B220$^+$ TLS adjacent to the meninges and in close proximity to the tumor tissue (dense nuclei, bottom) in GL261 tumor-bearing mice. The image is representative of 5 independent

animal experiments. Scale bar: 200 µm. **(b)** Schematic representation of TLS location in murine glioma models. TLS typically formed in the tumor-bearing hemisphere and were observed in multiple locations in close contact with the pia mater around the cortex or in the choroid plexuses. **(c)** $CD45^+B220^+$ TLS were not present in the brain of non-treated healthy mice or mice which had undergone mock tumor implantation followed by treatment with αCD40 antibodies. The images is representative of 3 mice. Scale bars: 200 µm. **(d-g)** Immunostaining of TLS in the brain of αCD40-treated GL261 tumor-bearing mice revealed the presence of (**d**) $B220^+IgG^+$ B cells and rare $B220^{low/-}IgG^+$ plasma cells (indicated by white arrows), (**e**) $F4/80^+$ macrophages, (**f**) rare GFP^+CD11c^+ dendritic cells, (**g**) $Foxp3^+$ T regs. The images are representative of 3 independent experiments. For clarity, the same area within the dotted square in (f) is shown on the bottom right of the panel in the absence of B220 and CD3 channels. The arrow indicates a $CD11c^+$ dendritic cell positive for GFP (or "tumor", white). The magenta arrows in (g) indicate cells that stained triple-positive for CD3, CD4 and FoxP3. Red arrows indicate cells that stained positive for CD3 only. Scale bars: 50µm. **(h-i)** Quantification of (**h**) the number of TLS per section and (**i**) TLS surface area plotted against the time of sacrifice for αCD40-treated GL261 tumor-bearing mice. Red circle indicates one αCD40-treated mouse. n(mice)=25; n(independent experiments)=3. **(j)** Representative images of dense $CD45^+B220^+$ clusters before (top panel) and after (bottom panel) laser capture microdissection. Scale bars: 50 µm. **(k)** Gene expression of *Ccl21* in laser capture micro-dissected $CD45^+B220^+$ clusters, compared with laser capture micro-dissected tumor tissue and normal brain tissue. n(LMD areas)=4/group. One way ANOVA with Tukey's multiple comparison correction. Black circle indicates TLS, black square indicates tumor and black triangle indicates healthy brain tissue. arb. units = arbitrary units. **(l-o)** Immunofluorescent stainings of αCD40-induced TLS in the CT-2A model showing TLS composition and organization. Arrows in (l) indicate a majority of $Ki67^+$ T cells (white arrows) and one $Ki67^+$ B cell (yellow arrow). The images are representative of two independent experiments and 4 mice. Scale bars: 50 µm. For all graphs in this figure, Bars: mean±SEM. Source data are provided as a Source Data file.

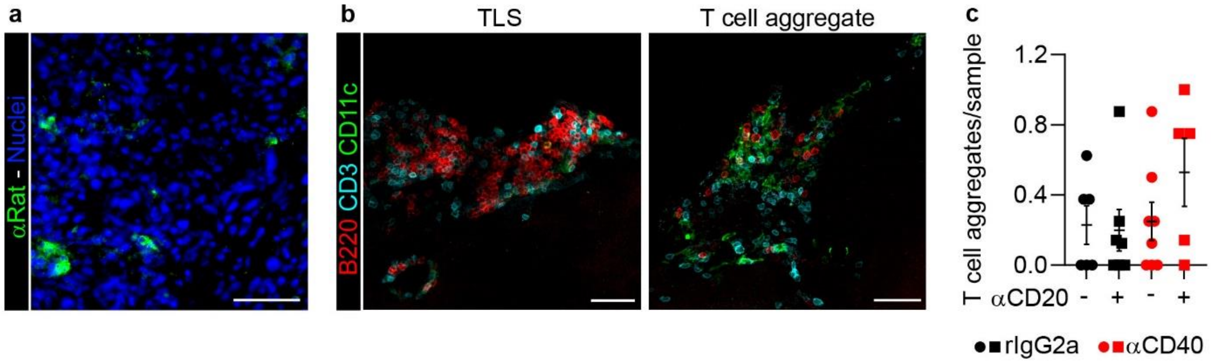

Supplementary Fig. 2: B cells were necessary for αCD40-mediated TLS induction.

(a) Immunofluorescent staining showing cells in the tumor area which are positive for the therapeutic αCD40 antibody. **(b)** Representative images showing the difference in composition between a TLS and a T cell aggregate in the meninges. TLS showed a clear B cell core surrounded by fewer T cells and scattered DCs, while T cell aggregates were infiltrated with only few B cells and comprised of a clear network of DCs. Scale bars: 50 µm. **(c)** Quantification of aggregates of T cells close to the meningeal layer in the indicated treatment groups. n(rIGg2a)=6mice; n(rIgG2a+αCD20)=7mice; n(αCD40)=8mice; n(αCD40+αCD20)=5mice. One way ANOVA with Tukey multiple correction. Bars: mean±SEM. Black circle indicates rat IgG2a (rIgG2a), black square indicates rIgG2a+αCD20, red circle indicates agonistic CD40 antibodies (αCD40), red square indicates αCD40+αCD20. Source data are provided as a Source Data file.

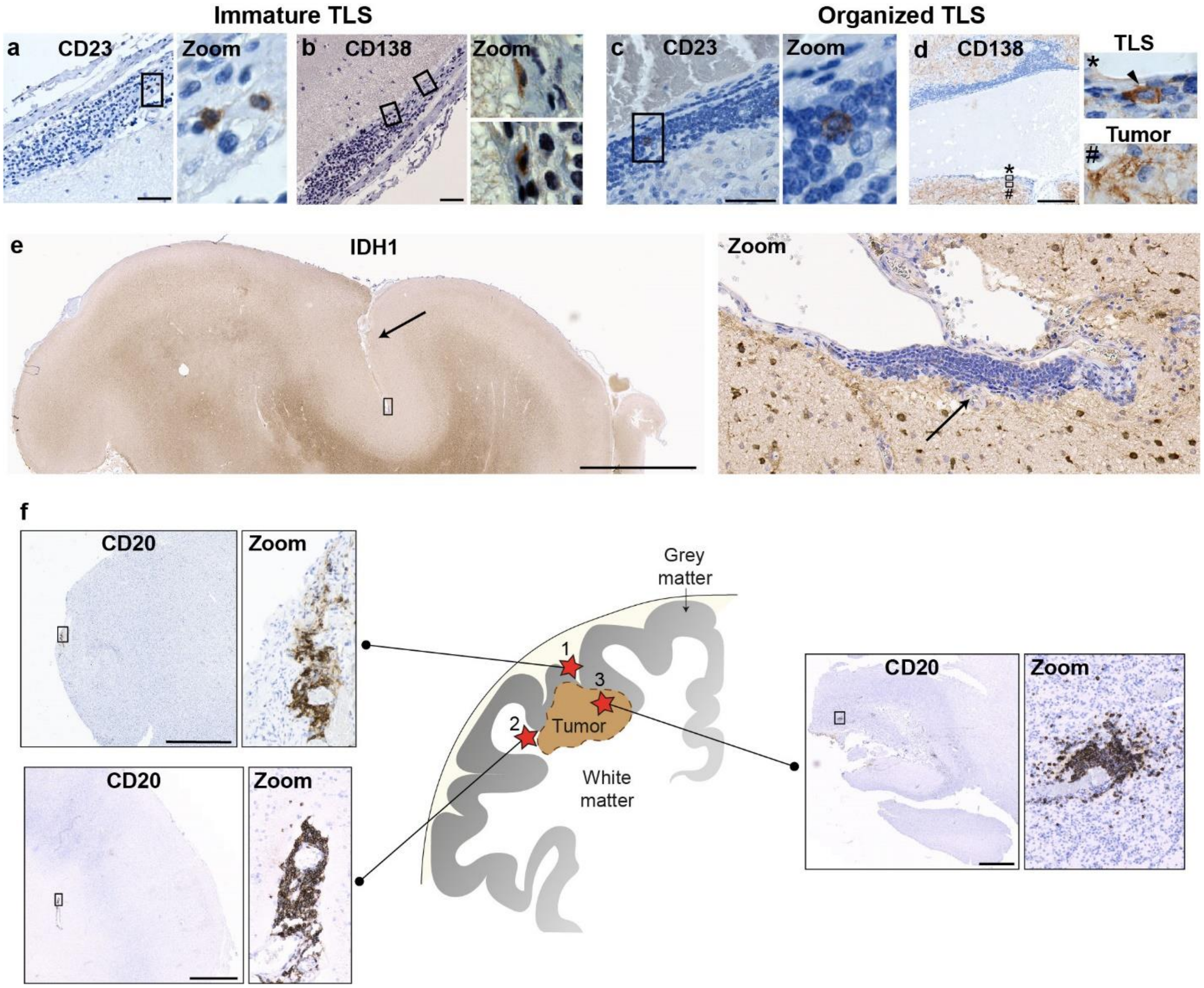

Supplementary Fig. 3: TLS composition and location in glioma patients.

(a-d) Immunohistochemical staining showing the presence of rare (**a,c**) $CD23^+$ follicular B cells and (**b,d**) $CD138^+$ plasma cells in immature and organized TLS. Stainings were performed on one representative immature TLS and one representative organized TLS. Zoom areas in (a-d) are indicated by black squares and the relative zoom panels are shown to the right of each image. The zoom panel indicated by an asterisk shows a $CD138^+$ plasma cell inside the TLS. The zoom panel indicated by a hash shows $CD138^{int}$ glioma cells in the tumor area. Scale bars: (a-c) 50µm, (d) 200 µm. **(e)** A dense cluster of nuclei in the depth of a cerebral sulcus, identified in a section obtained from a WHO Grade II oligodendroglioma (patient 6, Supplementary Table 1) that stained positive for mutated isocitrate dehydrogenase 1 (IDH1). The black square area in (e) is magnified to the right. Arrow in (e): cerebral sulcus. Arrow in zoom panel: dense cluster of nuclei. Scale bar: 5 mm. Stainings were performed in 4 patients. **(f)** Schematic representation of the locations were TLS were observed in human glioma samples: 1) in close proximity to the meninges, 2) in the white matter, close to the tumor and 3) inside the tumor tissue. For each location, a representative immunohistochemical staining is shown. This includes an overview of the tissue, where TLS location is indicated by a black square. A zoom of the square area is shown to the right. Scale bars: 2 mm. Stainings were performed in 26 patients.

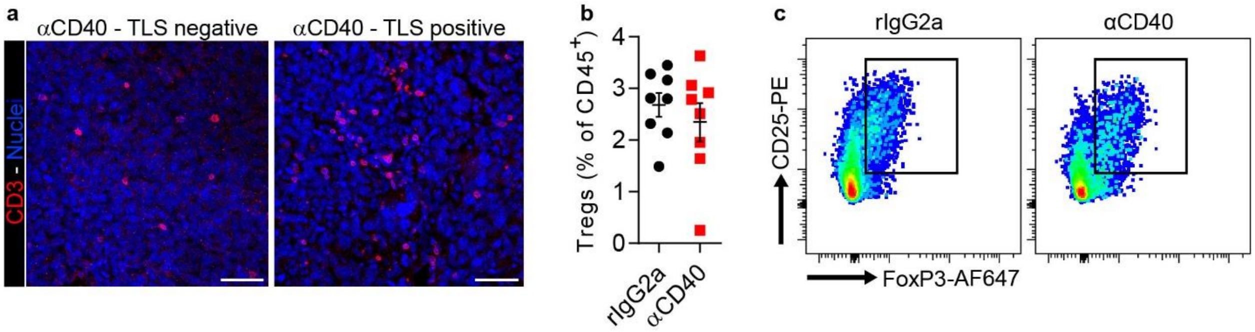

Supplementary Fig. 4: T cell characterization in αCD40-treated mice.

(a) Representative images of T cell infiltration in αCD40-treated GL261 tumors that stained positive or negative for TLS. Scale bar: 50 µm. **(b)** Quantification of $CD25^{+}FoxP3^{+}$ Tregs as a percentage of $CD45^{+}$ cells in GL261 tumors treated with rIgG2a or αCD40. n=8mice/group. Two-tailed *t*-test. Bars: mean±SEM. Black circle indicates rat rIgG2a (rIgG2a), red square indicates agonistic CD40 antibodies (αCD40). **(c)** Representative FACS plots of the $CD3^{+}CD4^{+}CD25^{+}FoxP3^{+}$ T cell population quantified in (b). Source data are provided as a Source Data file.

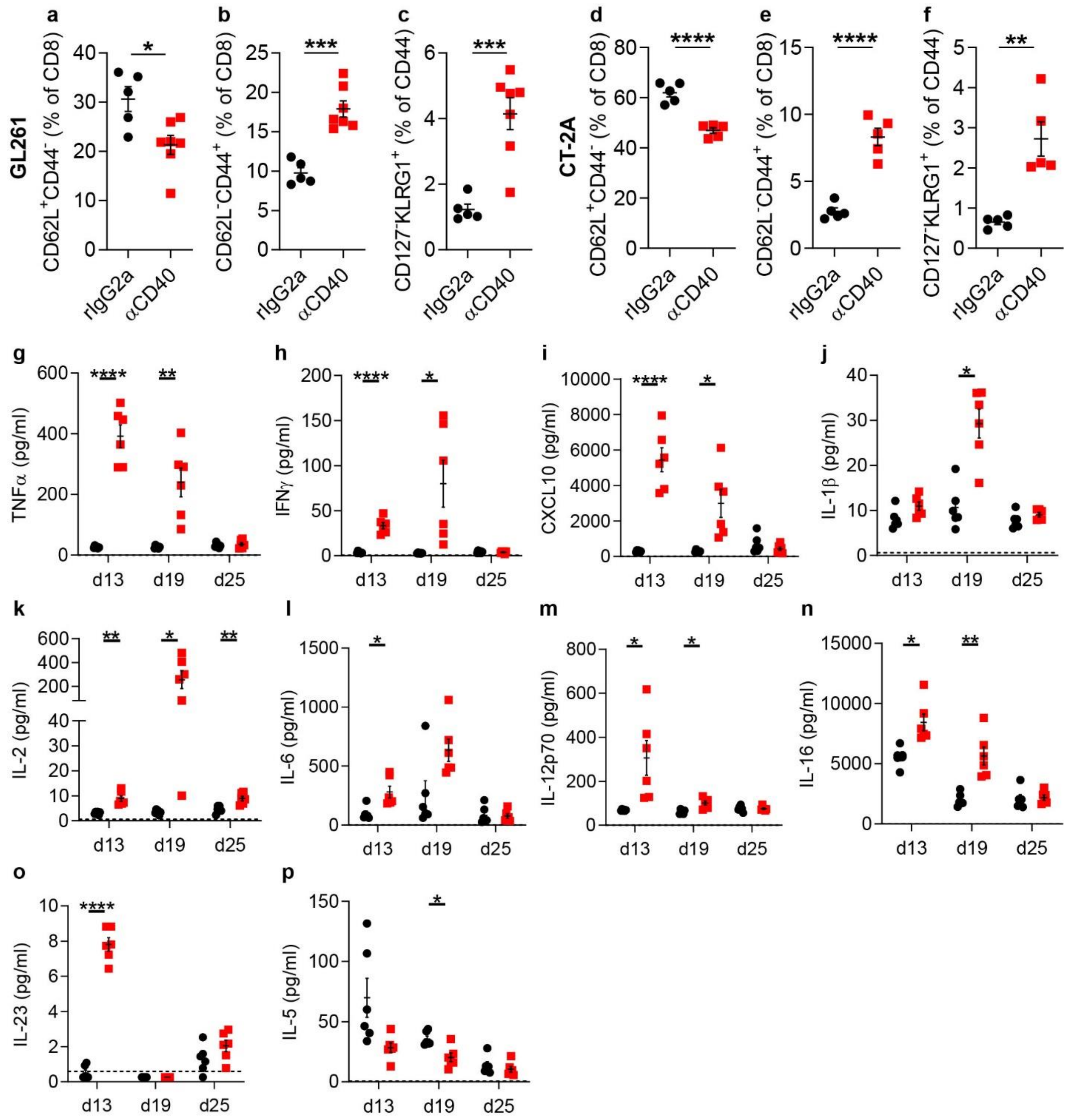

Supplementary Fig. 5: Systemic response to αCD40 therapy.

All data was obtained from mice that received rIgG2a or αCD40 antibodies on days 10, 13, 16 and 19 post-tumor implantation. **(a-f)** Quantifications obtained from spleens of GL261 and CT-2A tumor-bearing mice. n(GL261-rIgG2a)&(CT-2A)=5mice/group; n(GL261 αCD40)=7mice; Two tailed *t-test*. (**a,d**) Quantification of $CD62L^{+}CD44^{-}$ T cells as a percentage of $CD8^{+}$ cells. (**b,e**) Quantification of $CD62L^{-}CD44^{+}$ T cells as a percentage of $CD8^{+}$ cells. (**c,f**) Quantification of $CD127^{-}KLRG1^{+}$ cells as a percentage of $CD8^{+}CD44^{+}$ T cells. In (a) p=0.0135. In (b) p=0.0001. In (c) p=0.0007. In (d-e) p<0.0001. In (f) p=0.0014. **(g-q)** Systemic cytokine levels of (**g**) TNFα, (**h**) IFNγ, (**i**) CXCL10, (**j**) IL-1β, (**k**) IL-2, (**l**) IL-6, (**m**) IL-12p70, (**n**) IL-16, (**o**) IL-23 and (**p**) IL-5 in the serum of GL261 tumor-bearing mice on day 13, day 19 and day 25 post-tumor implantation. The dashed line indicates the lower limit of detection. n=6mice/group. Multiple *t*-test with Sidak-Bonferroni's correction for multiple comparisons. In (g) $p_{(d13)}<0.0001$¸ $p_{(d19)}=0.0035$. In (h)

$p_{(d13)}<0.0001$, $p_{(d19)}<0.0426$. In (i) $p_{(d13)}<0.0001$. $p_{(d19)}=0.0198$. In (j) $p_{(d19)}=0.0016$. In (k) $p_{(d13)}=0.0015$, $p_{(d19)}=0.0202$, $p_{(d25)}=0.0054$. In (l) $p_{(d13)}=0.0211$. In (m) $p_{(d13)}=0.0387$, $p_{(d19)}=0.0123$. In (n) $p_{(d13)}=0.0111$, $p_{(d19)}=0.0026$. In (o) $p_{(d13)}<0.0001$. In (p) $p_{(d19)}=0.0147$. For all graphs in this figure: Bars: mean±SEM. Black circle indicates rat IgG2a (rIgG2a), red square indicates agonistic CD40 antibodies (αCD40). *p<0.05, **p< 0.01, ***p < 0.001, ****p < 0.0001. Source data are provided as a Source Data file.

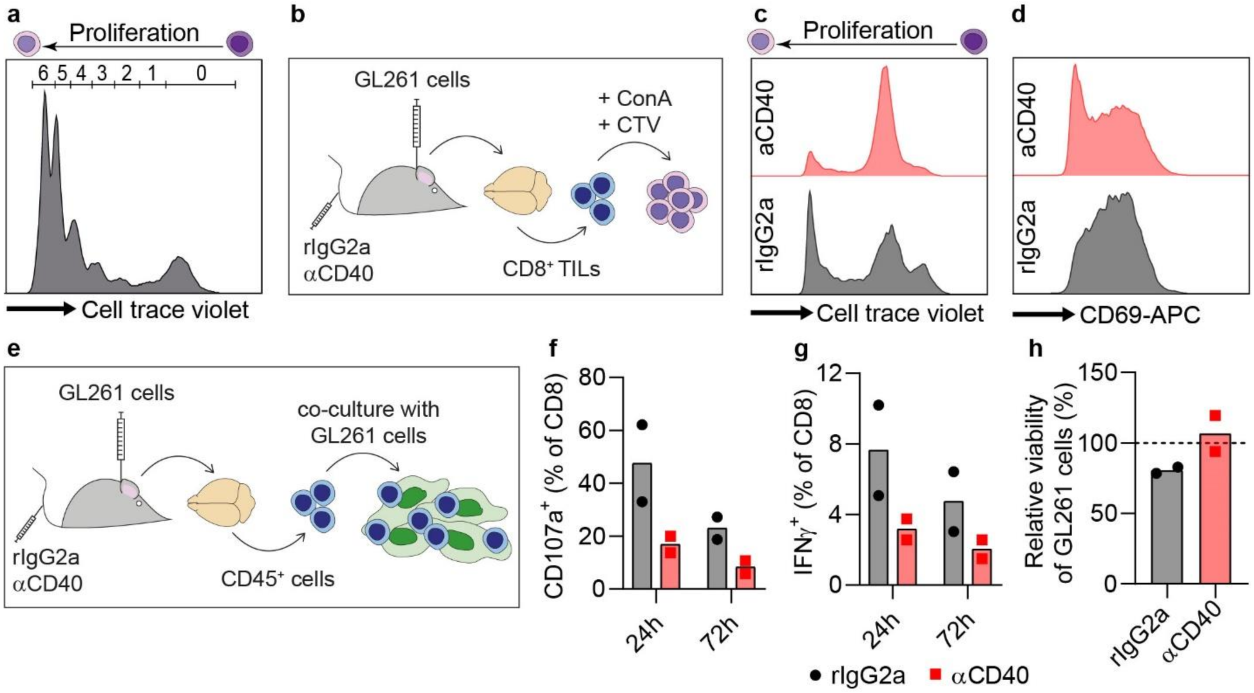

Supplementary Fig. 6: αCD40 induced a hypofunctional T cell phenotype in glioma-bearing mice.
(a) Example of how generations of proliferating CD8⁺ T splenocytes were defined based on cell trace violet staining. Each peak represents one generation. Generation 0 corresponds to the highest intensity peak, where cells did not divide. Generation 6 corresponds to the lowest intensity peak, where cells have divided roughly 6 times. **(b)** Schematic illustration of the experimental layout used to obtained data shown in panels (c-d). In brief, GL261 glioma-bearing mice were treated with rIgG2a or αCD40 antibodies on days 10, 13, 16 and 19 post-tumor implantation. On day 22, the mice were sacrificed and CD8⁺ T cells were isolated from the brain, stained with cell trace violet (CTV) to assess proliferation and re-stimulated *in vitro* with concanavalin A (ConA) for 24h and 72h. (**c**) Histogram showing the proportion of CD8⁺ T cells in each proliferation peak based on CTV staining. (**d**) Histogram showing CD69 protein levels on the CD8⁺ T cell population. (c-d) are representative of 2 mice/group. **(e)** Schematic illustration of the experimental layout used to obtained data shown in panels (f-h). In brief, GL261 glioma-bearing mice were treated with rIgG2a or αCD40 antibodies on days 10, 13, 16 and 19 post-tumor implantation. On day 22, the mice were sacrificed and CD45⁺ T cells were isolated from the brain and co-cultured with GL261 cells *in vitro* for 24 and 72h. **(f-g)** Percentages of (**f**) CD107a⁺ and (**g**) IFNγ⁺ CD8⁺ T cells in rIgG2a and αCD40 groups at the indicated time-points. **(h)** Relative viability of GL261 cells after 72h of co-culture. For all graphs in this figure: n=2mice/ group. Bars: mean. Black circle indicates rat IgG2a (rIgG2a), red square indicates agonistic CD40 antibodies (αCD40). Source data are provided as a Source Data file.

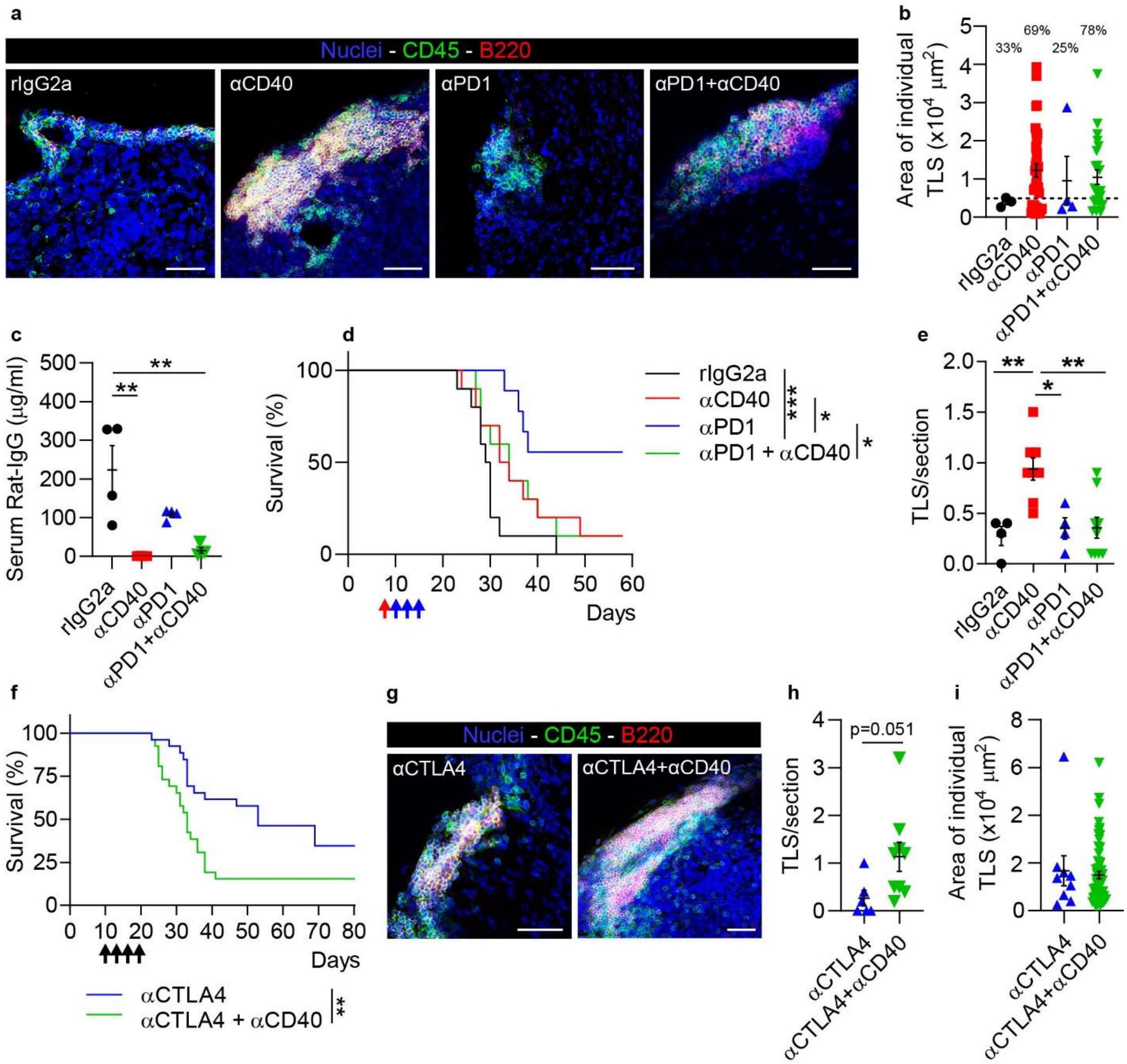

Supplementary Fig. 7: αCD40 impairs the response to PD-1 and CTLA-4 checkpoint blockade.

All data shown in this figure was obtained from GL261 glioma-bearing mice. In all panels except (d-e), αCD40 was administered in a sequential treatment regimen (on days 10, 13, 16 and 19). **(a)** Representative image of TLS in the indicated treatment groups. Scale bar: 50µm. **(b)** Quantification of the $CD45^+$ surface area of each individual TLS for the indicated treatment groups. Data points indicate individual TLS areas. The dotted line indicates the mean+SD of the rIgG2a group. Percentages indicate the proportion of TLS that were larger than the mean+SD of the rIgG2a group in each treatment group. n(rIgG2a)=3 TLS; n(αCD40)=36 TLS; n(αPD1)=4 TLS; n(αCD40+αPD1)=23 TLS. **(c)** Serum levels of rat antibodies (Rat IgG) in the indicated treatment groups on day 19 post-tumor implantation. n=4mice/group. $p_{(rIgG2a\ vs\ \alpha CD40)}$=0.0016, $p_{(rIgG2a\ vs\ \alpha PD1+\alpha CD40)}$=0.0027. One way ANOVA with Tukey's multiple correction. **(d)** Kaplan–Meier survival curve of mice treated with a single dose of αCD40 on day 9 or treated with a single dose of αCD40 on day 9 followed by three doses of αPD1 antibodies on days 10, 13 and 16 (as indicated by red and blue arrows, respectively). n(rIgG2a)&(αCD40)&(αPD1+αCD40)=10mice/group. n(αPD1)=9mice. $p_{(rIgG2a\ vs\ \alpha PD1)}$=0.0003, $p_{(\alpha CD40\ vs\ \alpha PD1)}$=0.0263, $p_{(\alpha PD1\ vs\ \alpha PD1+\ \alpha CD40)}$=0.0358. Log rank test. **(e)** Quantification of the number of TLS for the indicated treatment groups. n(rIgG2a)&(αPD1)=4mice; n(αCD40)=8mice; n(αPD1+αCD40)=9mice. $p_{(rIgG2a\ vs}$

$_{\alpha CD40)}$=0.005, $p_{(\alpha CD40\ vs\ \alpha PD1)}$=0.0135, $p_{(\alpha CD40\ vs\ \alpha PD1+\alpha CD40)}$=0.0019. One way ANOVA with Tukey's multiple correction. **(f)** Kaplan–Meier survival curve of mice treated with αCTLA-4 antibodies and/or αCD40 antibodies on days 10, 13, 16 and 19 (as indicated by arrows). n=26mice/group.p=0.0019. Log rank test; . **(g)** Representative image of TLS in the indicated treatment groups. Scale bar: 50µm. **(h,i)** Quantification of (**h**) the number and (**i**) the $CD45^+$ surface area of each individual TLS for the indicated treatment groups. Two-tailed *t*-test. In (h) n(αCTLA-4)=6mice; n(αCTLA-4+αCD40)=9mice. In (i) n(αCTLA-4)=9 TLS, n(αCTLA-4+αCD40)=61 TLS. In (b-c) and (e), black circle indicates rat IgG2a (rIgG2a), red square indicates agonistic CD40 antibodies (αCD40), blue triangle (pointing up) indicates PD1 blocking antibodies (αPD1), green triangle (pointing down) indicates αPD1+αCD40. In (h-i) blue triangle (pointing up) indicates CTLA-4 blocking antibodies (αCTLA-4), green triangle (pointing down) indicates αCTLA-4+αCD40. For all graphs in this figure, *p<0.05, **p< 0.01, ***p < 0.001. Bars: mean±SEM. Source data are provided as a Source Data file.

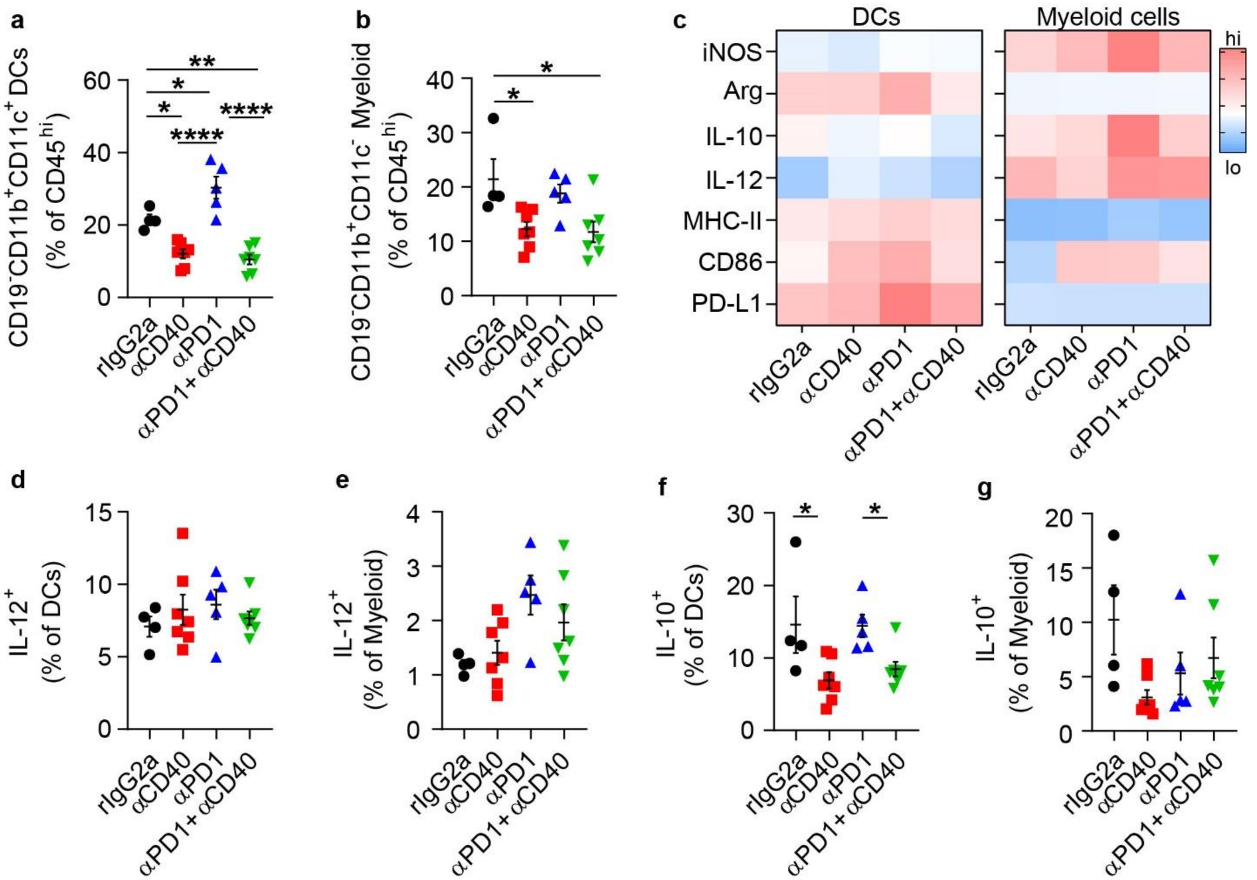

Supplementary Fig. 8: αCD40 activated brain-infiltrating dendritic cells and myeloid cells.

Phenotypic analysis of myeloid subtypes in GL261 glioma-bearing mice. **(a,b)** Quantification of (**a**) $CD19^-CD11b^+CD11c^+$ dendritic cells (DCs) and (**b**) $CD19^-CD11b^+CD11c^-$ myeloid cells as a percentage of immune cells in the brain of mice in the indicated treatment groups. In (a) $p_{(rIgG2a\ vs\ \alpha CD40)}=0.0108$, $p_{(rIgG2a\ vs\ \alpha PD1)}=0.0290$, $p_{(rIgG2a\ vs\ \alpha PD1+\alpha CD40)}=0.0030$, $p_{(\alpha CD40\ vs\ \alpha PD1)}<0.0001$, $p_{(\alpha PD1\ vs\ \alpha PD1+\alpha CD40)}<0.0001$. In (b) $p_{(rIgG2a\ vs\ \alpha CD40)}=0.0332$, $p_{(rIgG2a\ vs\ \alpha PD1+\alpha CD40)}=0.0229$. **(c)** Heatmap showing the expression levels (z-score) of activation and immunosuppression markers on DCs and myeloid cells in the brain of mice in the indicated treatment groups. **(d-e)** Quantification of $IL\text{-}12^+$ cells as a percentage of (**d**) DCs and (**e**) myeloid cells in the brain of mice in the indicated treatment groups. **(f-g)** Quantification of $IL\text{-}10^+$ cells as a percentage of (**f**) DCs and (**g**) myeloid cells in the brain of mice in the indicated treatment groups. In (f) $p_{(rIgG2a\ vs\ \alpha CD40)}=0.0382$, $p_{(\alpha CD40\ vs\ \alpha PD1)}=0.0287$. For all graphs in this figure, n(rIgG2a)=4mice; n(αCD40)&(αPD1+αCD40)=7mice; n(αPD1)=5mice. One way ANOVA with Tukey's multiple correction. *p<0.05, **p<0.01, ****p<0,0001. Bars: mean±SEM. Black circle indicates rat IgG2a (rIgG2a), red square indicates agonistic CD40 antibodies (αCD40), blue triangle (pointing up) indicates PD1 blocking antibodies (αPD1), green triangle (pointing down) indicates αPD1+αCD40. Source data are provided as a Source Data file.

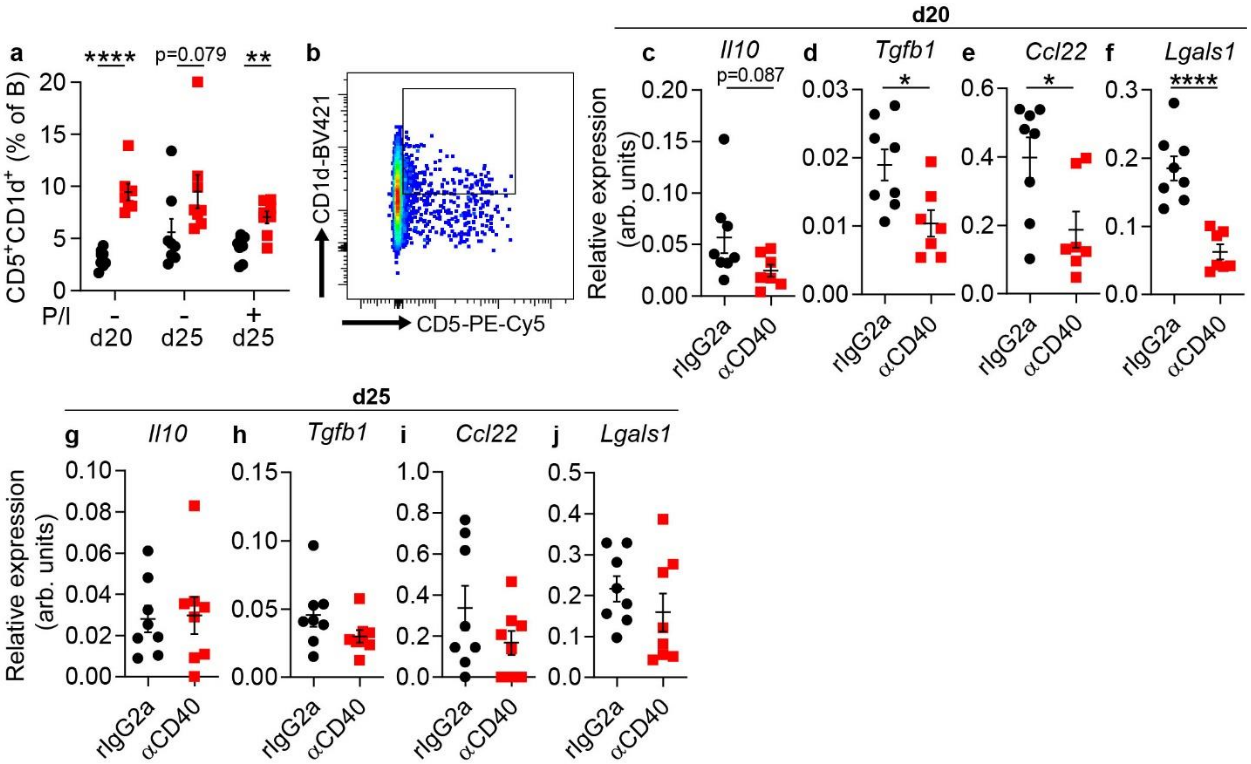

Supplementary Fig. 9: αCD40 therapy did not increase the expression of soluble immunosuppressive factors in B cells. Analysis of regulatory molecules on B cells in GL261 tumors. **(a)** CD5$^+$CD1d$^+$ cells as a percentage of CD19$^+$B220$^+$ B cells on day 20 and on day 25 post-tumor implantation, with or without 5h stimulation with PMA and Ionomycin (P/I). n=8mice/group in all groups except n(αCD40-d20)=7mice. $p_{(d20)}<0.0001$, $p_{(d25+P/I)}=0.0012$. Two-tailed *t*-test. **(b)** Representative plots of the CD1d$^+$CD5$^+$ B cell population quantified in (a). **(c-f)** Gene expression of *Il10, Tgfb*1, *Ccl22* and *Lgals1* on B cells sorted on day 20 post-tumor implantation. n(rIgG2a)=8mice; n(αCD40)=7mice. In (d) p=0.0145. In (e) p=0.0216. In (f) p<0.0001. **(g-j)** Gene expression of *Il10, Tgfb*1, *Ccl22* and *Lgals1* on B cells sorted on day 25 post-tumor implantation. n=8mice/group. (**c-j**) arb. units = arbitrary units. Two-tailed *t*-test. For all graphs in this figure: *p<0.05, **p<0.01, ***p<0.001, ****p<0.0001. Bars: mean±SEM. Black circle indicates rat IgG2a (rIgG2a), red square indicates agonistic CD40 antibodies (αCD40). Source data are provided as a Source Data file.

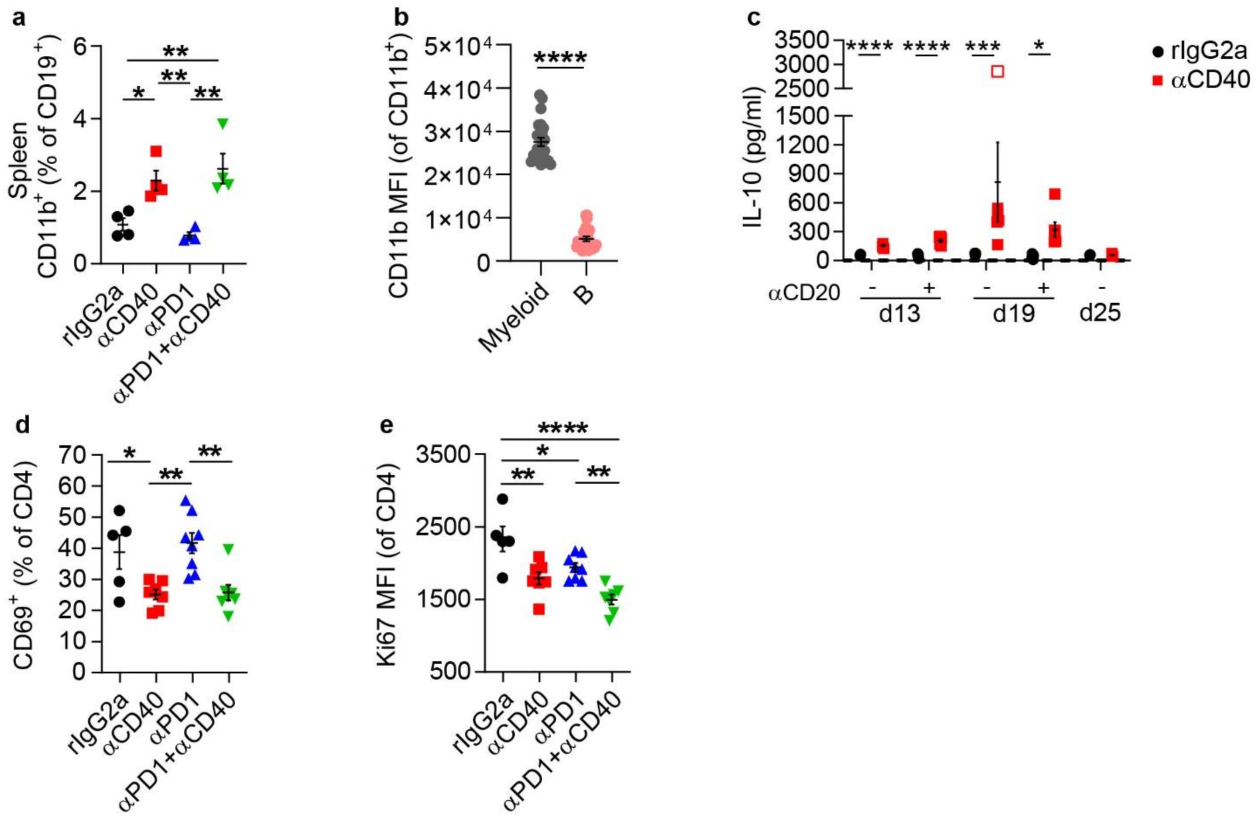

Supplementary Fig. 10: αCD40 therapy induced CD11b⁺ B cells and suppressed CD4⁺ T cells.

All figure panels show data from GL261 glioma-bearing mice. **(a)** Quantification of $CD11b^+$ cells as a percentage of B cells in the spleen, in the indicated treatment groups. n=4mice/group. $p_{(rIgG2a\ vs\ \alpha CD40)}=0.0334$, $p_{(rIgG2a\ vs\ \alpha PD1+\alpha CD40)}=0.0073$, $p_{(\alpha CD40\ vs\ \alpha PD1)}=0.0083$, $p_{(\alpha PD1\ vs\ \alpha PD1+\alpha CD40)}=0.0019$. One way ANOVA with Tukey's correction for multiple comparisons. **(b)** Mean fluorescence intensity (MFI) of CD11b on $CD11b^+$ myeloid cells versus $CD11b^+$ B cells in the brain. Data were merged from rIgG2a, αCD40, αPD1 and αCD40+αPD1 treatment groups. n=23mice. $p<0.0001$. Two-tailed *t*-test. **(c)** Systemic levels of IL-10 on days 13, 19 and 25 post-tumor implantation, after treatment with rIgG2a or αCD40 antibodies, with or without B cell depletion using an αCD20 antibody. The dashed line indicates the lower limit of detection. The data point marked as an empty red square was determined to be an outlier using the Grubbs test, thus it was excluded from the statistical analysis. n=6mice/group. $p_{(d13\text{-}\alpha CD20)}<0.0001$, $p_{(d13+\alpha CD20)}<0.0001$; $p_{(d19\text{-}\alpha CD20)}=0.0012$; $p_{(d19+\alpha CD20)}=0.0250$. Multiple *t*-test with Sidak-Bonferroni correction for multiple comparisons. **(d)** Quantification of $CD69^+$ cells as a percentage of $CD4^+$ T cells in the brain, in the indicated treatment groups. $p_{(rIgG2a\ vs\ \alpha CD40)}=0.0434$, $p_{(\alpha CD40\ vs\ \alpha PD1)}=0.0039$; $p_{(\alpha PD1\ vs\ \alpha PD1+\alpha CD40)}=0.0056$. **(e)** Mean fluorescence intensity (MFI) of Ki67 in $CD4^+$ T cells in the brain, in the indicated treatment groups. $p_{(rIgG2a\ vs\ \alpha CD40)}=0.0039$, $p_{(rIgG2a\ vs\ \alpha PD1)}=0.0395$, $p_{(rIgG2a\ vs\ \alpha PD1+\alpha CD40)}<0.0001$, $p_{(\alpha PD1\ vs\ \alpha PD1+\alpha CD40)}=0.0074$. In (d-e) n(rIgG2a)=5mice; n(αCD40)&(αPD1+αCD40)=7mice; n(αPD1)=8mice. One way ANOVA with Tukey's correction for multiple comparisons. For all graphs in this figure: *p<0.05, **p<0.01, ***p<0.001, ****p<0.0001. Bars: mean±SEM. Black circle indicates rat IgG2a (rIgG2a), red square indicates agonistic CD40 antibodies (αCD40), blue triangle (pointing up) indicates PD1 blocking antibodies (αPD1), green triangle (pointing down) indicates αPD1+αCD40, grey circle indicates myeloid cells (Myeloid), light pink circle indicates B cells (B). Source data are provided as a Source Data file.

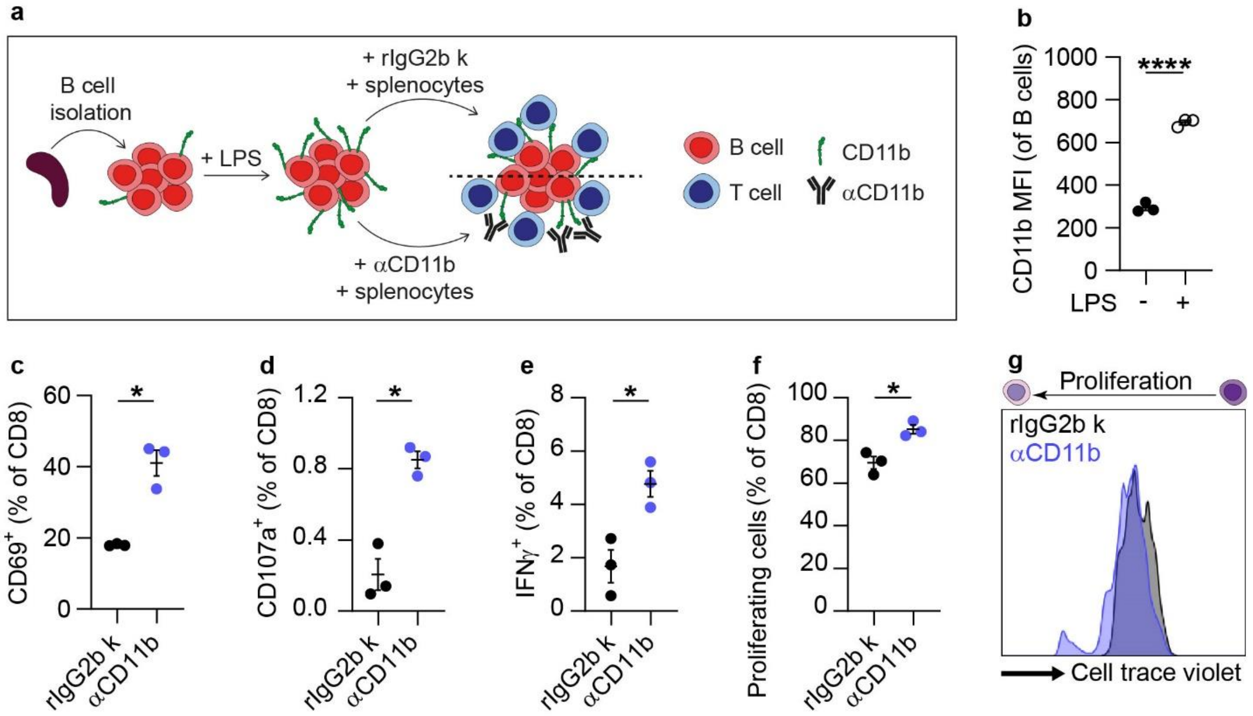

Supplementary Fig. 11: CD11b expression on B cells inhibits CD8+ T cell responses.

(a) Schematic illustration of the experimental layout used to obtained data shown in panels (b-g). In brief, B cells were isolated from wt C57BL/6 mice and co-cultured with LPS for 48h to induce upregulation of CD11b. LPS-treated B cells were then co-cultured for 72h with splenocytes in the presence of a control antibody (rIgG2b k) or a CD11b neutralizing antibody. Data on B and T cells were collected by FACS. **(b)** Mean fluorescence intensity (MFI) of CD11b on B cells with (black circle) or without (empty circle) LPS stimulation. $p<0.0001$. **(c-f)** Quantification of (**c**) CD69+ ($p=0.0264$), (**d**) CD107a+ ($p=0.0219$) (**e**) IFNγ+ ($p=0.0369$) and (**f**) proliferating cells as a percentage of CD8+ T cells after co-culture with LPS-induced B cells in the presence of rIgG2b k or an αCD11b antibody. $p=0.0323$. In (c-f), black circle indicates rat IgG2b k (rIgG2b k), purple circle indicates αCD11b neutralizing antibody (αCD11b). **(g)** Representative histogram showing the shift of proliferation peaks on CD8+ T cells co-cultured with B cells in the presence of a control antibody (rIgG2b k) or of a CD11b-neutralizing antibody. For all graphs in this figure: n=3mice/group. Two-tailed *t*-test; $*p<0.05$, $****p<0.0001$. Bars: mean±SEM. Source data are provided as a Source Data file.

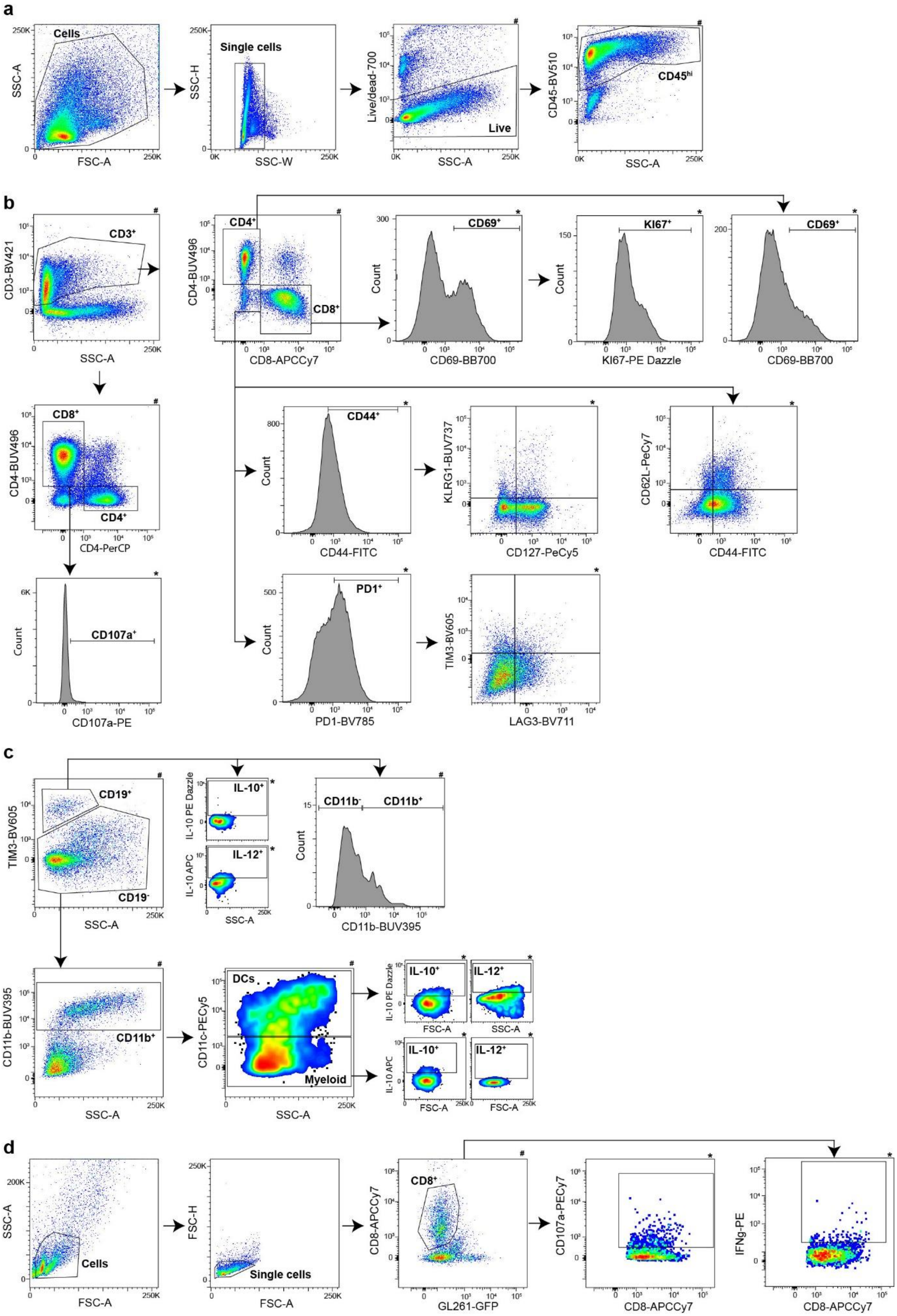
a
Cells
SSC-A
FSC-A
Single cells
SSC-H
SSC-W
Live/dead-700
Live
SSC-A
CD45-BV510
CD45hi
b
CD3-BV421
CD3+
SSC-A
CD4-BUV496
CD4+
CD8+
CD8-APCCy7
CD69+
Count
CD69-BB700
KI67+
KI67-PE Dazzle
CD69+
CD69-BB700
CD4-BUV496
CD8+
CD4+
CD4-PerCP
CD44+
CD44-FITC
KLRG1-BUV737
CD127-PeCy5
CD62L-PeCy7
CD44-FITC
CD107a+
CD107a-PE
PD1+
PD1-BV785
TIM3-BV605
LAG3-BV711
c
TIM3-BV605
CD19+
CD19-
SSC-A
IL-10 PE Dazzle
IL-10+
IL-10 APC
IL-12+
SSC-A
CD11b-
CD11b+
Count
CD11b-BUV395
CD11b-BUV395
CD11b+
SSC-A
CD11c-PECy5
DCs
Myeloid
SSC-A
IL-10 PE Dazzle
IL-10+
IL-12+
FSC-A
SSC-A
IL-10 APC
IL-10+
IL-12+
FSC-A
FSC-A
d
SSC-A
Cells
FSC-A
FSC-H
Single cells
FSC-A
CD8-APCCy7
CD8+
GL261-GFP
CD107a-PECy7
CD8-APCCy7
IFNg-PE
CD8-APCCy7

Supplementary Fig. 12: Gating strategy used for flow cytometry analysis of brain-infiltrating immune cells.

(a) Gating strategy used to select live $CD45^{high}$ immune cells from CD45-enriched single cell suspensions obtained from tumor-bearing brains in Fig. 2l-n, 4d-n, 5a-b, 5e-j, 6a-d, 6h-j and Supp. Fig. 4b-c, 8a-g, 9a-b, 10b, 10d-e. **(b)** Gating strategy used to identify $CD3^+$ T cells, $CD8^+$ T cells, $CD44^+CD62L^-$ CD8 T cells, $CD127^-KLRG1^+$ CD8 T cells, $CD69^+$ CD8 T cells, $CD107a^+$ CD8 T cells and $PD1^+TIM3^+LAG3^+$ CD8 T cells in Fig. 4d-n, and $CD3^+$ T cells, $CD8^+$ T cells and $CD69^+Ki67^+$ CD8 T cells in Fig. 5a-b, 5e-j, and $CD8^+$, $CD4^+$ T cells in Fig 6i-j, and $CD4^+$ T cells, $CD69^+$ CD4 T cells in Supp. Fig. 10d-e. **(c)** Gating strategy used to identify $CD19^+$ B cells, IL-10^+ B cells, IL-12^+ B cells and $CD11b^+$ B cells in Fig. 6a-d, and $CD19^-CD11b^+CD11c^+$ DCs, $CD19^-CD11b^+CD11c^-$ myeloid cells, IL-10^+ DCs or myeloid cells, IL-12^+ DCs or myeloid cells in Supp. Fig 4a-g, and $CD11b^+$ B cells and $CD11b^+$ myeloid cells in Supp. Fig 10b. **(d)** Gating strategy used to identify $CD8^+$ T cells, $CD107a^+$ CD8 T cells, $IFN\gamma^+$ CD8 T cells in Supp. Fig 6b-g. Asterisk indicates gates based on fluorescent minus one (FMO) controls. Hash indicates gates based on unstained controls.

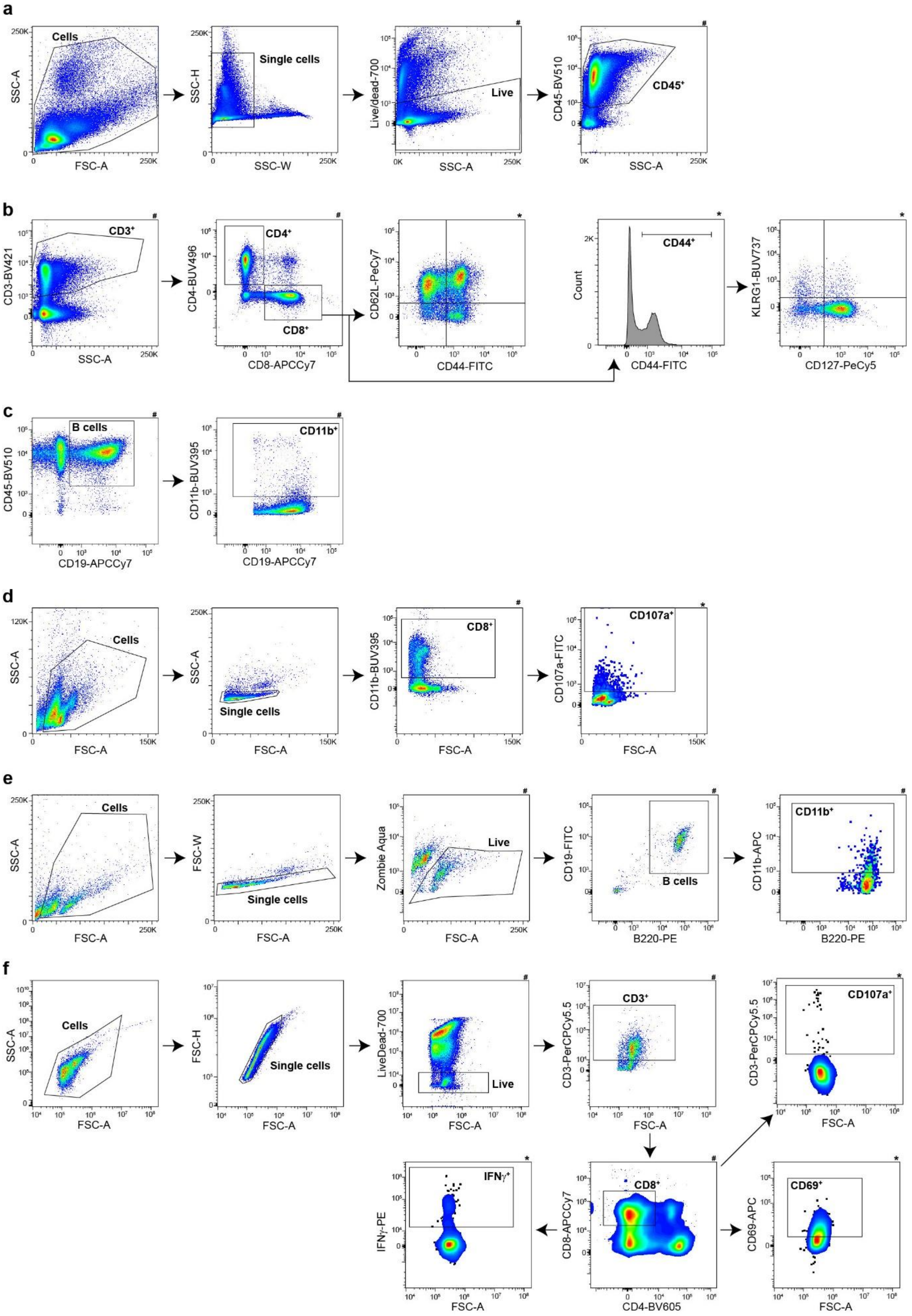
a
Cells
SSC-A
FSC-A
Single cells
SSC-H
SSC-W
Live
Live/dead-700
CD45⁺
CD45-BV510
b
CD3⁺
CD3-BV421
CD4⁺
CD8⁺
CD4-BUV496
CD8-APCCy7
CD62L-PeCy7
CD44-FITC
CD44⁺
Count
KLRG1-BUV737
CD127-PeCy5
c
B cells
CD45-BV510
CD19-APCCy7
CD11b⁺
CD11b-BUV395
d
CD8⁺
CD11b-BUV395
CD107a⁺
CD107a-FITC
e
FSC-W
Zombie Aqua
CD19-FITC
B220-PE
CD11b-APC
f
FSC-H
LiveDead-700
CD3-PerCPCy5.5
CD3-PerCPCy5.5
CD8-APCCy7
CD4-BV605
IFNγ⁺
IFNγ-PE
CD69⁺
CD69-APC

Supplementary Fig. 13: Gating strategy used for flow cytometry analysis of splenocytes.

(a) Gating strategy used to select live $CD45^+$ immune cells from single cell suspensions obtained from spleens in Supp. Fig. 5a-f, 10a. **(b)** Gating strategy used to identify $CD62L^+CD44^-$ CD8 T cells, $CD62L^-CD44^+$ CD8 T cells, $CD127^-$ $KLRG1^+$ CD8 T cells in Supp. Fig. 5a-f. **(c)** Gating strategy used to identify $CD11b^+$ B cells in Supp. Fig. 10a. **(d)** Gating strategy used to identify $CD8^+$ T cells and $CD107a^+$ CD8 T cells in Fig. 4o-r. **(e)** Gating strategy used to identify $CD11b^+$ B cells in Supp. Fig. 11b. **(f)** Gating strategy used to identify $CD69^+$ CD8 T cells, $CD107a^+$ CD8 T cells, $IFN\gamma^+$ CD8 T cells in Supp. Fig. 11c-e. Asterisk indicates gates based on fluorescent minus one (FMO) controls. Hash indicates gates based on unstained controls.
