# Supplementary material for: Agonistic CD40 therapy induces tertiary lymphoid structures but impairs responses to checkpoint blockade in glioma
Source: Nat Commun. 2021 Jul 5;12:4127. doi: 10.1038/s41467-021-24347-7 (PMC8257767; doi:10.1038/s41467-021-24347-7)
Supplement: Supplementary file 2 — Descriptions of Additional Supplementary Files [file 41467_2021_24347_MOESM2_ESM.pdf]

Descriptions of Additional Supplementary Files

**Supplementary Movie 1**
**Description:** Z-stack of a tertiary lymphoid structure in the brain of a GL261 glioma-bearing mouse. Green: CD45; Red: B220; Blue: Nuclei.
