# Supplementary material for: Agonistic CD40 therapy induces tertiary lymphoid structures but impairs responses to checkpoint blockade in glioma
Source: Nat Commun. 2021 Jul 5;12:4127. doi: 10.1038/s41467-021-24347-7 (PMC8257767; doi:10.1038/s41467-021-24347-7)
Supplement: Supplementary file 4 — Reporting Summary [file 41467_2021_24347_MOESM4_ESM.pdf]

nature research

Corresponding author(s): Anna Dimberg

Last updated by author(s): Jun 2, 2021

Reporting Summary

Nature Research wishes to improve the reproducibility of the work that we publish. This form provides structure for consistency and transparency in reporting. For further information on Nature Research policies, see Authors & Referees and the Editorial Policy Checklist .

Statistics

For all statistical analyses, confirm that the following items are present in the figure legend, table legend, main text, or Methods section.

n/a	Confirmed	
[ ]	[x]	The exact sample size (*n*) for each experimental group/condition, given as a discrete number and unit of measurement
[ ]	[x]	A statement on whether measurements were taken from distinct samples or whether the same sample was measured repeatedly
[ ]	[x]	The statistical test(s) used AND whether they are one- or two-sided<br>*Only common tests should be described solely by name; describe more complex techniques in the Methods section.*
[x]	[ ]	A description of all covariates tested
[ ]	[x]	A description of any assumptions or corrections, such as tests of normality and adjustment for multiple comparisons
[ ]	[x]	A full description of the statistical parameters including central tendency (e.g. means) or other basic estimates (e.g. regression coefficient) AND variation (e.g. standard deviation) or associated estimates of uncertainty (e.g. confidence intervals)
[ ]	[x]	For null hypothesis testing, the test statistic (e.g. *F*, *t*, *r*) with confidence intervals, effect sizes, degrees of freedom and *P* value noted<br>*Give P values as exact values whenever suitable.*
[x]	[ ]	For Bayesian analysis, information on the choice of priors and Markov chain Monte Carlo settings
[ ]	[x]	For hierarchical and complex designs, identification of the appropriate level for tests and full reporting of outcomes
[x]	[ ]	Estimates of effect sizes (e.g. Cohen's *d*, Pearson's *r*), indicating how they were calculated

Our web collection on statistics for biologists contains articles on many of the points above.

Software and code

Policy information about availability of computer code

Data collection	For flow cytometry: BD FACSDiva 8.0.2, CytExpert 2.4. For microscopy: Leica Application Suite X 3.0.1.15878, Zen Blue 1.1.2.0. For gene expression: QuantStudio 3 v1.4.3. For serum cytokines: Discovery Workbench 4.0.12
Data analysis	For flow cytometry: FlowJo v10.5.3, Cytosplore 2.2.1. For image analysis: ImageJ v1.51, Leica Application Suite X 3.6.0.20104; ImageScope v12.3.3.5048, QuPath v0.2.0. For cytokine analysis: Discovery Workbench 4.0.12

For manuscripts utilizing custom algorithms or software that are central to the research but not yet described in published literature, software must be made available to editors/reviewers. We strongly encourage code deposition in a community repository (e.g. GitHub). See the Nature Research guidelines for submitting code & software for further information.

Data

Policy information about availability of data

All manuscripts must include a data availability statement. This statement should provide the following information, where applicable:

- Accession codes, unique identifiers, or web links for publicly available datasets
- A list of figures that have associated raw data
- A description of any restrictions on data availability

Source data are provided with this paper.

Field-specific reporting

Please select the one below that is the best fit for your research. If you are not sure, read the appropriate sections before making your selection.

[x] Life sciences [ ] Behavioural & social sciences [ ] Ecological, evolutionary & environmental sciences

Life sciences study design

All studies must disclose on these points even when the disclosure is negative.

Sample size	No sample size calculation was performed to predetermine sample size in either in vivo/ex vivo or in vitro experiments. Sample size for in vivo/ex vivo experiments was determined based on previous experience with biological variation in the in vivo models used (PMID: 26363010, 26645582) and accounting for the 3R principle. The same applies to in vitro experiments, which were conducted with murine cells freshly isolated from naive mice. We believe that the chosen sample size is sufficient since our results are reproducible and consistent with respect to statistical differences between the treatment groups.
Data exclusions	For in vivo experiments, mice were excluded from survival analysis if they died or were sacrificed for reasons other than brain tumor growth e.g. a few mice got an adverse reaction in direct association with the fourth administration of agonistic CD40 mAb. For flow cytometry analysis, samples were excluded if too few events were recorded for reliable analysis.
Replication	In vivo studies and in vitro assays were repeated multiple times (at least two independent experiments) and by different scientists to ensure reproducibility. The manuscript only includes the data which was reproducible.
Randomization	For in vivo/ex vivo studies, allocation of mice to each treatment group was random. Mice of similar ages were randomly divided in treatment groups at the start of each experiment. Since the mice were of similar age and background, further randomization was not necessary. For in vitro assays testing the effect of agonistic CD40 mAb or CD11b neutralizing mAb, randomization was not necessary, since cells derived from the same mice were divided in two groups and treated with either the aforementioned antibodies or with the respective isotype controls.
Blinding	The investigators were not blinded during group allocation, treatments and data collection, because there were rare side effects related to repeated administration of agonistic CD40 mAbs that needed to be carefully monitored. However, the investigators were blinded during data analysis.

Reporting for specific materials, systems and methods

We require information from authors about some types of materials, experimental systems and methods used in many studies. Here, indicate whether each material, system or method listed is relevant to your study. If you are not sure if a list item applies to your research, read the appropriate section before selecting a response.

Materials & experimental systems

n/a	Involved in the study	
☐	☒	Antibodies
☐	☒	Eukaryotic cell lines
☒	☐	Palaeontology
☐	☒	Animals and other organisms
☐	☒	Human research participants
☒	☐	Clinical data

Methods

n/a	Involved in the study	
☒	☐	ChIP-seq
☐	☒	Flow cytometry
☒	☐	MRI-based neuroimaging

Antibodies

Antibodies used	All antibodies used in this study for immunofluorescence staining or flow cytometry are listed in Supplementary table 3, Supplementary table 4 and Supplementary table 5. Information regarding fluorochromes, clone, dilution, catalog number and company are also reported in the above mentioned Supplementary tables.<br><br>In addition, the following antibodies were used for in vivo or in vitro studies:<br>- CD11b neutralization assay: αCD11b neutralizing antibody (clone M1/70, 10 µg/ml, Biolegend, San Diego, CA, USA) or a control rIgG2b k antibody (clone RTK4530, 10 µg/ml, Biolegend).<br>- In vivo antibodies: agonistic rat-anti-mouse CD40 (clone: FGK4.5, Cat# BE0016), rat-anti-mouse PD-1 (clone: RMP1-14, Cat# BE0146), mouse-anti-mouse CTLA-4 (clone 9D9, Cat# BP0164), rat-anti-mouse CD20 (clone AISB12, Cat# BE0302) from Bioxcell.
Validation	Relevant publication or validations assays for all antibodies used for immunofluorescence staining or flow cytometry can be found on the RRID portal (https://scicrunch.org/resources) or on the manufacturer's website. For easy access to this information, the research resource identifiers (RRIDs) and catalogue numbers of all antibodies used for are provided in Supplementary table 3, Supplementary table 4 and Supplementary table 5.<br><br>In addition:<br>- we validated all antibodies used for immunofluorescence by staining fresh frozen lymph nodes or spleens collected from C57BL/6 mice, and verified their specificity. Similarly, all antibodies used for flow cytometry were validated by staining splenocytes collected from C57BL/6 mice (as positive control), and verified with respective fluorescence minus one (FMO) control stainings.<br>- all antibodies used for IHC on human tissue were validated by using human lymphoid tissue as positive control with each

staining. RRIDs have been indicated when applicable in the Methods section.

Therapeutic antibodies:
- Agonistic rat-anti-mouse CD40 (clone: FGK4.5, Cat# BE0016, 100 μg/dose) has been previously used in several studies PMID: 23455713, 22156349, 26437243
- rat-anti-mouse PD-1 (clone: RMP1-14, Cat# BE0146, 200 μg/dose) has been previously used in several studies PMID: 27775706, 25754329, 23873688
- mouse-anti-mouse CTLA-4 (clone 9D9, Cat# BP0164, 100 μg/dose) has been previously used in several studies PMID: 25142145, 25623164, 24367702
- rat-anti-mouse CD20 (clone AISB12, Cat# BE0302, 200μg/dose) has been previously used in several studies PMID: 20561083

Neutralizing antibody
CD11b neutralization assay: αCD11b antibody (clone M1/70, 10 μg/ml, Biolegend, San Diego, CA, USA) has been previously used for neutralization assays in several studies PIMD: 17003381, 26207521

Eukaryotic cell lines

Policy information about cell lines

Cell line source(s)

GL261: gift from Dr. Geza Safrany, NRIRR, Budapest, Hungary. The cell line was well characterized by Dr. Geza and the details can be found in the reference, PMID: 16734735.

CT-2A: gift from T. Seyfried, Boston College, Boston, MA, USA. The cell line was established by Dr. Seyfried and the details can be found in the following reference, PMID: 8297420.

Authentication

The cell lines used in this study were not authenticated.

Mycoplasma contamination

The cell lines routinely tested negative for mycoplasma contamination.

Commonly misidentified lines
(See ICLAC register)

No misidentified cell lines were used in this study.

Animals and other organisms

Policy information about studies involving animals; ARRIVE guidelines recommended for reporting animal research

Laboratory animals

This study involved laboratory animals. Species: Mus musculus. Strain: C57BL6. Gender: female. Age: six to ten-week-old.

Wild animals

This study did not involve wild animals.

Field-collected samples

This study did not involve the use of field-collected samples

Ethics oversight

All animal experiments were approved by the Uppsala County regional ethics committee (permits C1/14, C26/15, N164/15 and 5.8.18-19429/2019), and were performed according to the guidelines for animal experimentation and welfare of Uppsala University.

Note that full information on the approval of the study protocol must also be provided in the manuscript.

Human research participants

Policy information about studies involving human research participants

Population characteristics

The histological and molecular tumor characteristics of the cohorts are shown in Supplementary Table 1. No additional patients were recruited to the study. Glioma samples from treatment-naïve patients containing meningeal tissue were selected from biobanks and anonymized. This part of the study was authorized by the regional Ethics Committee of Uppsala, Sweden (DNR 2010/291).

Recruitment

No additional participants were recruited to the study. Glioma samples from treatment-naïve patients containing meningeal tissues were selected from biobanked material. This selection was considered necessary since our observations from mouse models indicated that tertiary lymphoid structures were mainly present in meningeal regions. Results from the histological analysis were not associated with any clinical data or survival data, and correlated only to the presence of tertiary lymphoid structures and the amount of T cells infiltrating the tumor. It is possible that tertiary lymphoid structures were present in meningeal regions in other parts of the brain that have not been included in our examination. Nevertheless, our results give a clear indication that the prevalence of TLS, and the likelihood of identifying these structures in a given tissue, is associated with the number of enhanced T cells in the tumor.

Ethics oversight

The study was approved by the institutional review board at the Uppsala University Hospital, Uppsala, Sweden (DNR2010/05), the regional Ethics Committee of Uppsala, Sweden (DNR2015/089, DNR 2010/291) and the Ethics Committee of Western Sweden (EPN/DNR: 559-12). A statement that all participation was based on informed consent has been added to the Methods section.

Note that full information on the approval of the study protocol must also be provided in the manuscript.

Flow Cytometry

Plots

Confirm that:

- [x] The axis labels state the marker and fluorochrome used (e.g. CD4-FITC).
- [x] The axis scales are clearly visible. Include numbers along axes only for bottom left plot of group (a 'group' is an analysis of identical markers).
- [x] All plots are contour plots with outliers or pseudocolor plots.
- [x] A numerical value for number of cells or percentage (with statistics) is provided.

Methodology

Sample preparation	Single cell suspensions of tumor-bearing brains were obtained by enzymatic dissociation of the whole brain (minus the cerebellum) using a gentleMACS Octo Dissociator and the Tumor Dissociation kit (Miltenyi Biotec, Bergisch Gladbach, Germany). Myelin depletion was achieved by either using Myelin Removal Beads II (Miltenyi Biotec) or by resuspending the cells in a solution of 25% BSA (in PBS) and centrifuging at 650xg for 20min on a low brake (brake =2) to separate the myelin ring from the cell pellet. CD45+ immune cells and CD8+ T cells were enriched using either Mouse CD45 MicroBeads (Miltenyi Biotec) or Mouse CD8 (TIL) MicroBeads (Miltenyi Biotec), respectively.<br><br>Spleens were mechanically dissociated and cranial lymph nodes were digested by using 2.0 Wunsch U/ml of liberase TL (Roche, Basel, Switzerland) for 20 min at 37°C. Lymph nodes and spleens were subsequently passed through a 70m strainer (Corning, Sigma-Aldrich, St. Louis, MO, USA) in PBS to obtain a single cell suspension.<br><br>After isolation, cells were stained using a live-dead dye (Supplementary Table 4 and 5) following the instructions of the manufacturer. Unspecific Fc receptor binding in all single-cell suspensions was blocked by using anti-mouse CD16/CD32 antibody (clone 93, Biolegend, San Diego, CA, USA). Cells were stained for the markers of interest using fluorochrome-conjugated antibodies (Supplementary Table 4 and 5). All antibodies were diluted from stock concentration according to the ratios reported in Supplementary Table 4 and 5. For staining of FoxP3, the FOXP3 Fix/Perm Buffer Set (BioLegend) was used following the instructions of the manufacturer. For intracellular cytokine staining, the eBioscience™ Invitrogen™ Intracellular Fixation & Permeabilization Buffer Set (Thermo Fisher Scientific, Waltham, MA, USA) was used following the instructions of the manufacturer.
Instrument	FACSCanto II; LSR Fortessa; FACS AriaIII (all from BD BioSciences), CytoFLEX LX(Beckman Coulter).
Software	Data collection: BD FACSDiva 8.0.2, CytExpert v0.2.0. Data analysis: FlowJo v10.5.3; Cytosplore 2.2.1.
Cell population abundance	B cells were sorted from spleens and lymph nodes selecting for CD45+CD19+B220+ single cells (Figure 4). The sorted cells were collected in RLT lysis buffer, thus a purity analysis of the samples post-sorting was not feasible.
Gating strategy	Doublets (based on FSC and SSC values) and dead cells (based on live/dead staining) were gated out from the starting cell population to select only live, single cells. Then, the main populations of interest were selected using dot plots (e.g. CD3+CD8+). Further analysis was performed on the selected gates to identify subpopulation of cells positive for the markers of interest using dot plots or histograms (e.g. the CD44+CD26L- subpopulations of CD3+CD8+ T cells or the CD1d+CD5+ subpopulation of CD19 +B220+ B cells). The boundary between positive and negative cell populations was defined using fluorescence minus one (FMO) controls and unstained controls. Gating strategies are shown in Supplementary Fig. 12.

- [x] Tick this box to confirm that a figure exemplifying the gating strategy is provided in the Supplementary Information.
